# Domain atrophy creates rare cases of functional partial protein domains

**DOI:** 10.1186/s13059-015-0655-8

**Published:** 2015-04-30

**Authors:** Ananth Prakash, Alex Bateman

**Affiliations:** European Molecular Biology Laboratory, European Bioinformatics Institute (EMBL-EBI), Wellcome Trust Genome Campus, Hinxton, Cambridgeshire, CB10 1SD UK

## Abstract

**Background:**

Protein domains display a range of structural diversity, with numerous additions and deletions of secondary structural elements between related domains. We have observed a small number of cases of surprising large-scale deletions of core elements of structural domains. We propose a new concept called domain atrophy, where protein domains lose a significant number of core structural elements.

**Results:**

Here, we implement a new pipeline to systematically identify new cases of domain atrophy across all known protein sequences. The output of this pipeline was carefully checked by hand, which filtered out partial domain instances that were unlikely to represent true domain atrophy due to misannotations or un-annotated sequence fragments. We identify 75 cases of domain atrophy, of which eight cases are found in a three-dimensional protein structure and 67 cases have been inferred based on mapping to a known homologous structure. Domains with structural variations include ancient folds such as the TIM-barrel and Rossmann folds. Most of these domains are observed to show structural loss that does not affect their functional sites.

**Conclusion:**

Our analysis has significantly increased the known cases of domain atrophy. We discuss specific instances of domain atrophy and see that there has often been a compensatory mechanism that helps to maintain the stability of the partial domain. Our study indicates that although domain atrophy is an extremely rare phenomenon, protein domains under certain circumstances can tolerate extreme mutations giving rise to partial, but functional, domains.

**Electronic supplementary material:**

The online version of this article (doi:10.1186/s13059-015-0655-8) contains supplementary material, which is available to authorized users.

## Background

Protein domains are independently evolving structural and function units. These units have been recombined in many ways creating proteins that perform diverse functions. Proteins, in the course of evolution, may acquire (domain gain) or lose (domain loss) one or more entire domains by a variety of means [1,2]. At a smaller scale of modification, protein domains can gain or lose secondary structural elements through insertion or deletion of amino acid residues. Insertions or deletions are known to significantly influence protein folding [3] and are considered important evolutionary factors which influence protein structure and function [4]. Proteins and their domains are only marginally stable, such that the mutation of a single residue may be sufficient to induce the unfolding of the domain. Single point mutations can lead to a large range of human diseases. However, through evolution a protein’s sequence is slowly changed so that quite divergent sequences can give rise to a similar three-dimensional (3D) structure. This means that proteins are tolerant of small stepwise changes in their sequence.

Analyses of a large number of domain structures have indicated significant variability in domain lengths [5]. These variations are mostly attributed to insertions or deletions in loops, coils or of a few secondary structural elements, leaving the domain core largely unaffected [5-7]. Cases of such domain length variations caused by gain of accessory secondary structural elements or ‘embellishments’ are well documented. Examples of the so-called domain elaborations can be observed in the HUP superfamily [8], wherein embellishments influence interactions, substrate binding and stability of the core domain.

**Figure 1:**
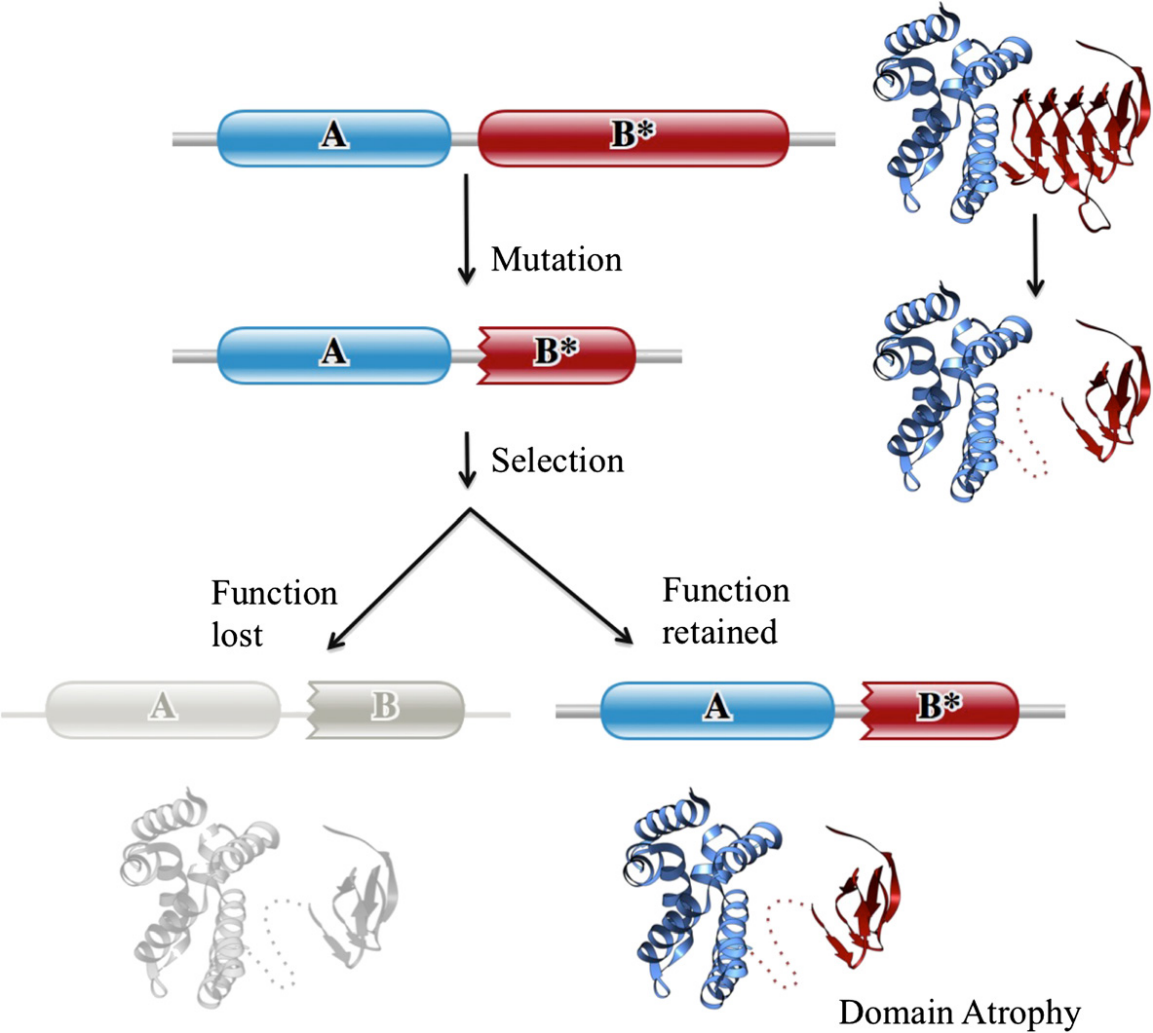
Schematic representation of domain atrophy event. A protein domain in a particular architecture undergoes truncation due to mutation. A protein with truncated domain that retains its functional role (structural or enzymatic) is positively selected, while a protein with non-viable truncated domain is lost. Complete and incomplete domain boundaries are denoted by smooth and toothed edges, respectively. The dotted line in the toy example at the right shows the region of upstream domain-bounded atrophy.

Similar to domain elaborations, one could envisage events leading to loss or degradation of a domain’s secondary structure elements necessitated by similar evolutionary constraints as above. We propose the term ‘domain atrophy’ for events that lead to significant loss of core secondary structure elements (Figure 1). Such loss of domain-structural elements has been observed in the ‘truncated globins’ [9], bacterial luciferases [10] and recently in a domain of unknown function DUF2172 [11].

It seems possible that the process of domain atrophy occurs by a single large-scale mutation, which raises the question of how can a protein tolerate such a large destabilising event. Such events of loss of >10 amino acid residues are more likely to be very destabilising. Losses at protein termini are more likely to be tolerated, particularly if they are of peripheral secondary structures. For example, partial deletion of the first seven residues at the N-terminus of Ig-domain of the human muscle protein titin only marginally decreased its stability by 2.8 kcal/mol [12], however mutations within the core are unlikely to be tolerated. Deletions of many amino acids are normally unlikely to be tolerated unless they are found in a loop region. Therefore, natural occurrences of stable protein domains with large-scale deletions of core secondary structures [10,11] are intriguing and suggest that compensatory mechanisms that help stabilise these atrophied domains must exist.

An alternative possibility is that a globular domain that undergoes an atrophy event then becomes disordered and may adopt some new function. We believe that we are unlikely to be able to observe such events as the pattern of conservation is likely to significantly change leading to very rapid sequence divergence. However, the relationship of domain atrophy to protein disorder may be an interesting avenue for future research.

Unlike domain elaboration, which is commonly seen in protein structures, structural data and literature on domain atrophy is very scarce. Using sequence based protein-HMM models of domain families, we have devised an algorithm to identify potential cases of domain atrophy and have identified several new cases in proteins of known structure. Where 3D structures of the atrophied domain were not available, we have used sequence mapping onto reference complete domain structures to infer potential domain atrophy events.

A companion paper by Triant and Pearson [13] describes several computational artefacts that can produce partial domain instances, which may confound the discovery of cases of domain atrophy. They show that domains can appear short because they have been broken up into pieces by the alignment process, or because they are annotated on incorrectly assembled proteins, or because the Pfam domain models have been built from two or more evolutionarily mobile domains. In this paper, we use several strategies to avoid these biological and computational artefacts, and identify a set of atrophied domains that are complementary to the ‘structural partials’ identified by Triant and Pearson.

## Results and discussion

In order to quantify the magnitude of structural loss we have defined a metric called the Atrophy Score (see Methods), which indicates the fraction of the domain that is likely to be missing. It is based on looking at matches of Pfam profile-HMMs to a sequence. For some domains only part of the profile-HMM model may match to the sequence. We use these partial matches to identify where a protein domain is potentially atrophied. The atrophy score is equivalent to the fraction of a Pfam profile-HMM model that is missing from a protein domain. While a positive atrophy score indicates structural loss, zero or negative atrophy scores indicate complete domain boundaries, that is, no atrophy. We did not investigate instances with an atrophy score below 0.15, where less than 15% of the domain is lost. Domains showing this small level of atrophy are very likely to be enriched in false positives where loss of peripheral secondary structures can be tolerated. A feature of the atrophy score is the possibility of scores to reach 1 or higher. This can happen when one domain is nested within another, leading to a negative inter-domain distance. These cases are excluded from our analysis.

A partial match to a Pfam profile-HMM is only weak evidence for domain atrophy. As Triant and Pearson show, even if a match to the whole HMM is not present one can often find that the similarity can be extended to the full domain length with a sequence similarity comparison. Therefore, we look in particular at partial domains that are end bounded by either another domain, or the terminus of the sequence.

**Figure 2:**
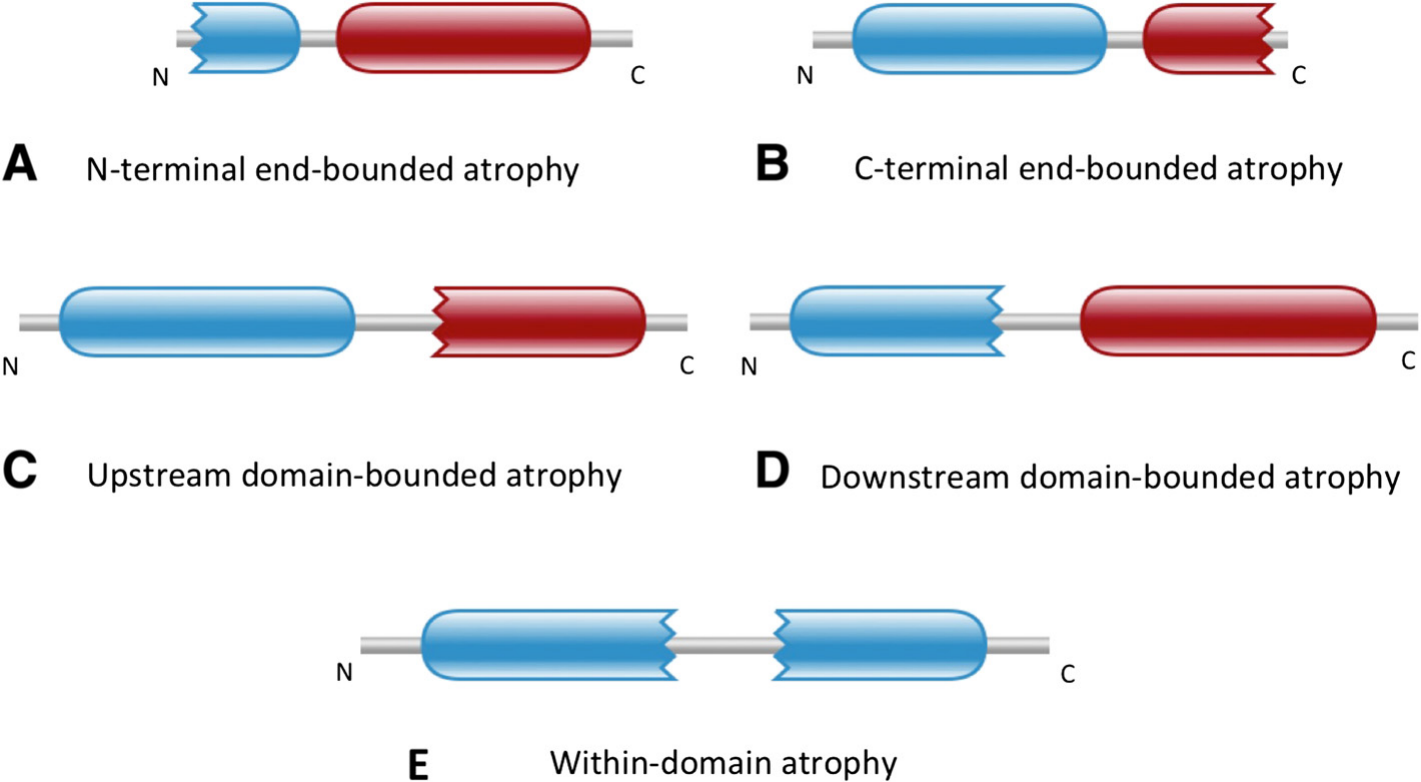
The five classes of domain atrophy events. Complete domains are represented with round-ended boundaries and incomplete domains are represented with jagged-ended boundaries.

We have categorised domain atrophy into five different types that are fully described in the Methods section (Figure 2). The five types are: (1) N-terminal end-bounded atrophy; (2) C-terminal end-bounded atrophy; (3) Upstream domain-bounded atrophy; (4) Downstream domain-bounded atrophy; and (v) Within-domain atrophy.

Domain instances that scored between 0.15 and 1 were manually examined to identify potential false positive cases (see Table 1). Manual inspection helped to discern probable true cases of domain atrophy from possible false positive cases. We were able to confirm eight cases, which have a known structure and a further 67 cases that belonged to a family where a homologous structure was known. In the following section we discuss some examples of domain atrophy that we have discovered. A full list of true and putative domain atrophy cases can be found in Additional file 1.

**Table 1:** **Manual classification of cases of domain atrophy**

		**N-terminal end-bounded atrophy**	**C-terminal end-bounded atrophy**	**Upstream domain-bounded atrophy**	**Downstream domain-bounded atrophy**	**Within-domain atrophy**
	**Total**	**499 (100%)**	**468 (100%)**	**125 (100%)**	**331 (100%)**	**213 (100%)**
Structure available	True domain atrophy	6 (1.20%)	0 (0.00%)	0 (0.00%)	2 (0.60%)	0 (0.00%)
	False positive	161 (32.26%)	119 (25.42%)	27 (21.60%)	104 (31.41%)	45 (21.13%)
Homologous structure available	Putative domain atrophy	34 (6.81%)	26 (5.55%)	0 (0.00%)	3 (0.90%)	4 (1.88%)
	False positive	216 (43.28%)	218 (46.58%)	91 (72.80%)	207 (62.53%)	99 (46.48%)
No structure available	Unknown	82 (16.43%)	105 (22.43%)	7 (5.60%)	15 (4.53%)	65 (30.52%)

The table shows the breakdown of domain atrophy instances with atrophy scores between 0.15 and 1.

### N-terminal end-bounded atrophy

#### Bacterial luciferase domain (Pfam: PF00296)

The bacterial luciferase domain of the non-fluorescent flavoprotein (NFP) from *Photobacterium phosphoreum* LuxF (UniProt: P12745), shows an atrophy score of 0.31, indicating a loss of nearly one-third of the domain’s structure.

The bacterial luciferase domain, homolog of bacterial luciferase subunits [14], is present mostly among members of gammaproteobacteria. NFP is thought to function as a ‘molecular sponge’ by sequestering myristylated flavine mononucleotide, the side-product of the bioluminescence pathway [14].

**Figure 3:**
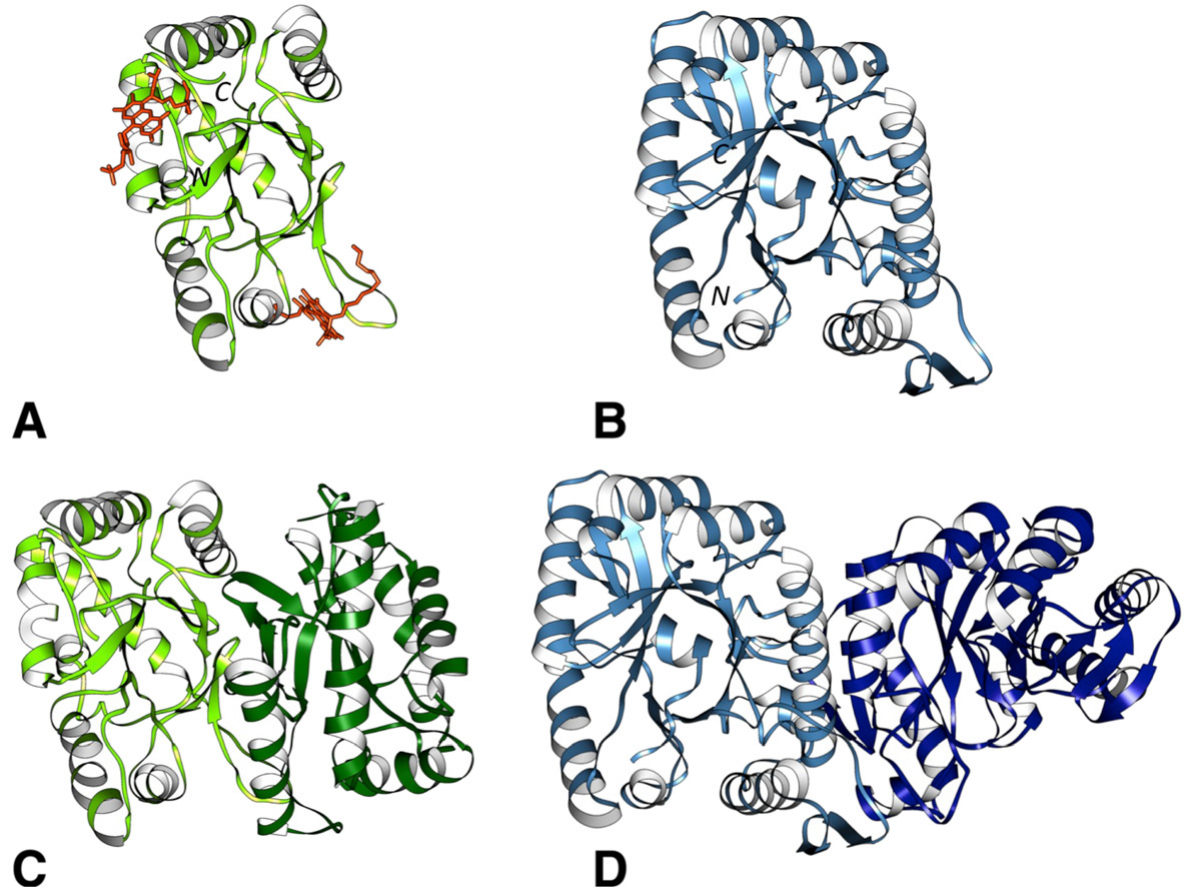
N-terminal end-bounded atrophy: Bacterial luciferase. **(A)** Monomer of the atrophied bacterial luciferase domain of *Photobacterium phosphoreum* non-fluorescent flavoprotein (PDB: 1FVP, light green) bound to ligand FMA (6-(3-tetradecanoic acid) flavine mononucleotide, orange). **(B)** Monomer of the *Bacillus cereus* reference structure (PDB: 2B81, light blue). **(C)** Homo-dimer complex of 1FVP. The exposed hydrophobic core due to domain atrophy is stabilised by the new dimer interface. **(D)** Homo-dimer complex of 2B81 showing dimerisation on the same side of the molecule.

NFP of *P. phosphoreum* (PDB: 1FVP) resembles a partial TIM-barrel-like fold with one β-strand and three α-helices missing [15] (Figure 3A). The complete structure of the bacterial luciferase domain from *Bacillus cereus* (PDB: 2B81) was chosen as the reference domain for comparison. The reference domain has a (β/α)_8_ TIM-barrel fold (Figure 3B) with a characteristic β-barrel structure consisting of eight alternating β-strands and α-helices.

Although identified as N-terminal end-bounded atrophy, comparison with the reference domain (PDB: 2B81) shows that β_1_, α_1_ and β_2_ of 2B81 structurally aligns with the corresponding structural elements at the N-terminal region of 1FVP. However α_2_, β_3_, α_3_ and α_4_, of the reference domain 2B81 (residues 61-125, 132-192), have no corresponding matching structural elements on 1FVP. This suggests that *P. phosphoreum* bacterial luciferase domain is an example of within-domain atrophy rather than N-terminal end-bounded atrophy as first thought. We also identified a similar case of domain atrophy in the orthologous protein of *Photobacterium leiognathi* (UniProt: P09142, PDB: 1NFP).

**Figure 4:**
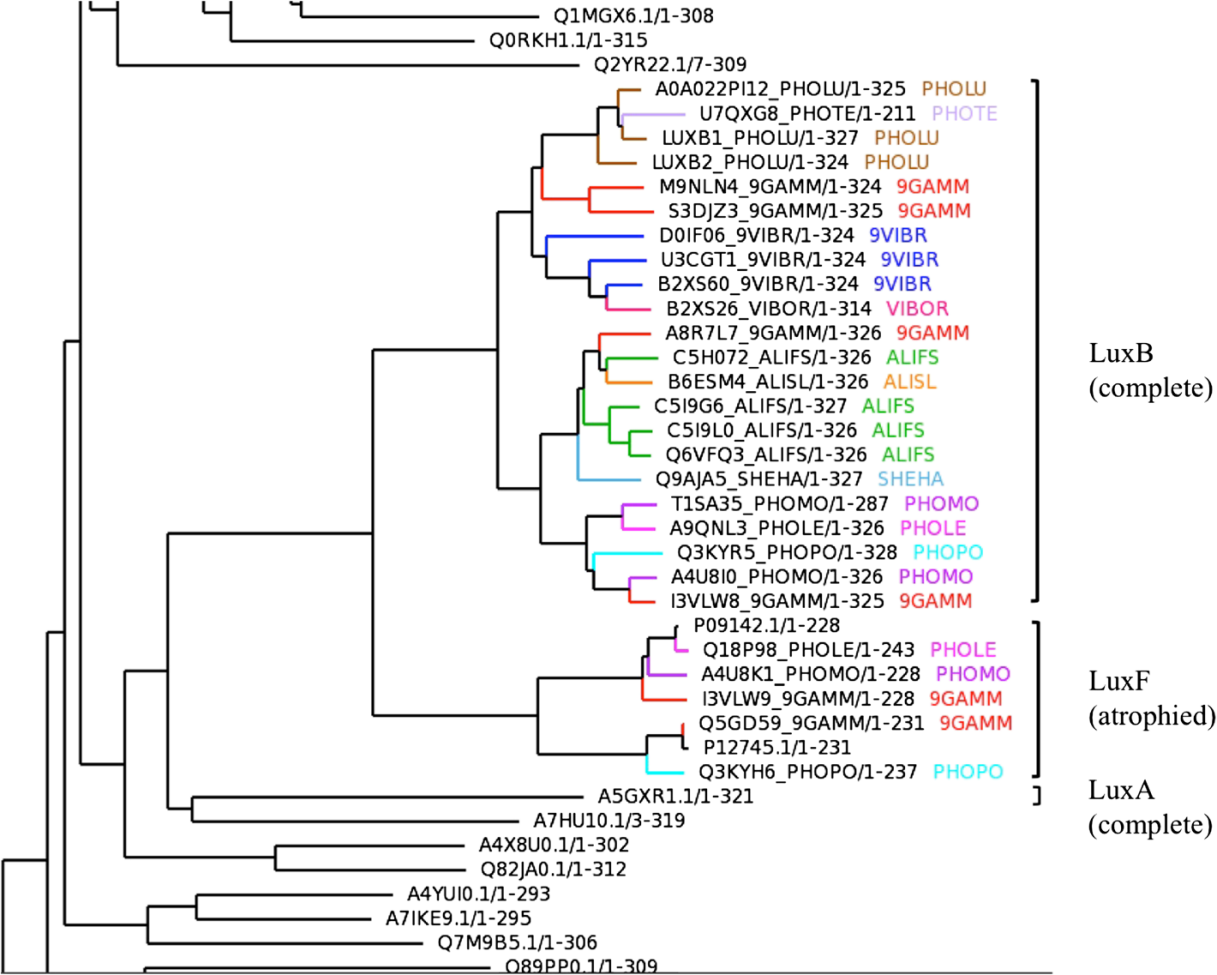
A section of the phylogenetic tree of bacterial luciferase domains. The bacterial luciferase, non-fluorescent flavoprotein (LuxF) and the alkanal monooxygenase beta (LuxB) share a common ancestor. The ancestral fold of luciferase is a complete TIM-barrel fold observed in LuxB and LuxA proteins.

A key issue is in determining whether domain atrophy has occurred is whether the ancestral fold of bacterial luciferase domains was indeed a TIM-barrel. Phylogenetic analysis of bacterial luciferase family indicates that the LuxF protein clade is completely enclosed by LuxB (Figure 4) and that the ancestral fold must be the complete TIM-barrel fold observed in the LuxB and LuxA proteins.

Does the structure shed any light on how an atrophied domain can tolerate a large deletion? The TIM-barrel fold has a central hydrophobic β-barrel surrounded by alpha helices, which shield the barrel from the solvent. However, the monomeric structure of the atrophied bacterial luciferase domain shows a barrel with a large hydrophobic cleft apparently exposed to the solvent. The atrophied domain buries this hydrophobic surface by forming a homo-dimeric protein interaction interface (Figure 3C) [16,17]. The hydrophobic core residues thus exposed by the atrophy event are stabilised by interactions at the new dimer interface. We hypothesise that such a compensatory mechanism may be a crucial factor in the selection of the otherwise unstable atrophied domain. Interestingly bacterial luciferase, in its complete form [18], also functions as a dimer *in vivo* and forms a homo-dimer with the dimer interface being on the same side of the molecule as observed in the atrophied domain-dimers (Figure 3D).

#### AMP-binding domain (Pfam: PF00501)

**Figure 5:**
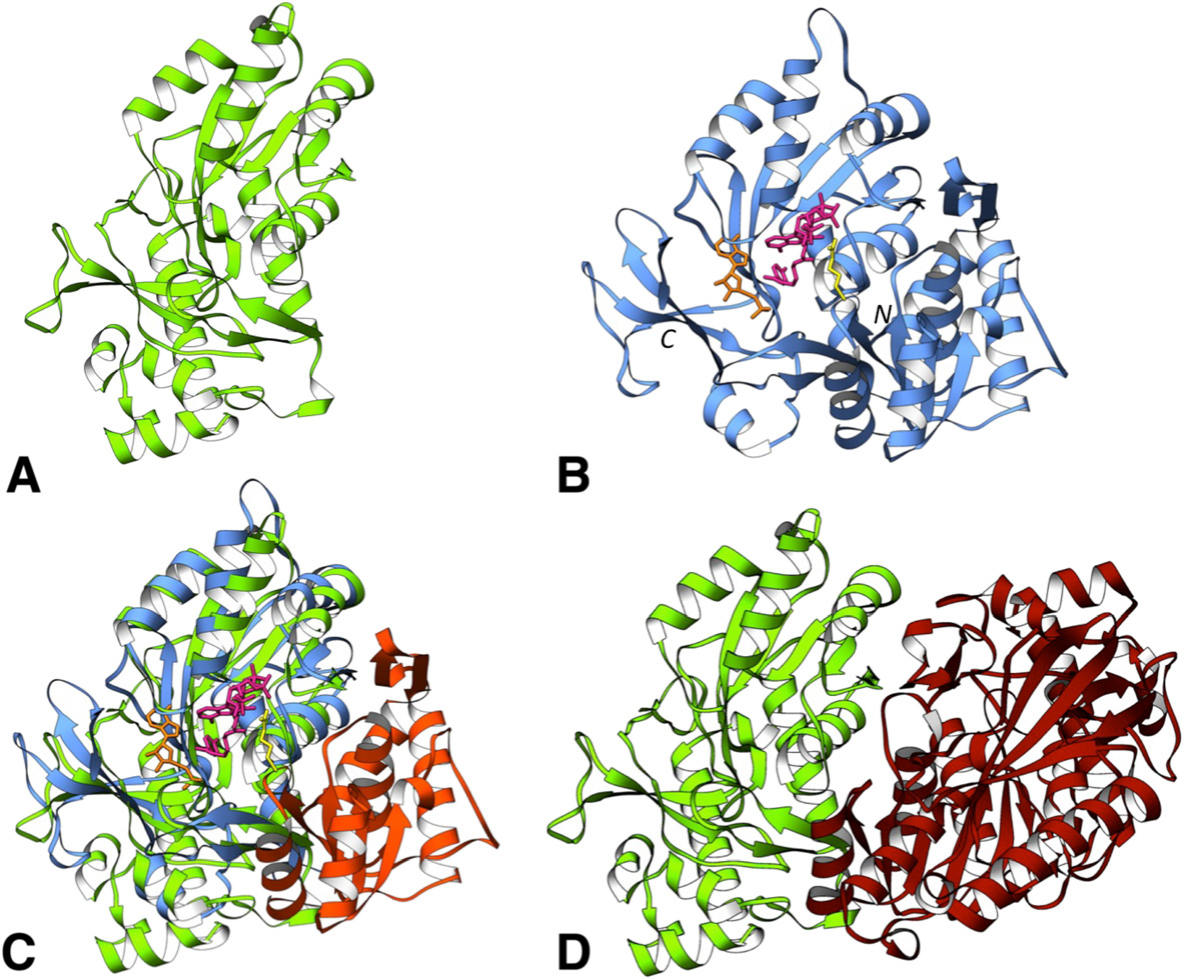
N-terminal end-bounded atrophy: AMP-binding domain. Comparison of **(A)** the atrophied N-terminal AMP-binding domain of *B. cenocepacia* phenylacetate-CoA ligase (PDB: 2Y27, green) and **(B)** reference structure, *Alcaligenes sp* 4-chlorobenzoyl CoA ligase (PDB: 3CW9, blue) bound to AMP (gold) and 4-Chlorophenacyl-coenzyme A (magenta). **(C)** Superposition of the N-terminal domains shows the atrophied region of 2Y27, which corresponds to the first sub-domain of 3CW9 (orange, residues 1-150). The residues of the first sub-domain, except Arg87 (yellow), have minimal interactions with ligands AMP (gold) or 4-Chlorophenacyl-coenzyme A. **(D)** Homo-dimeric complex of atrophied *B. cenocepacia* phenylacetate-CoA ligase forms an arrangement, which at the interface is structurally similar to the first-sub domain.

The Adenosine monophosphate (AMP) binding domain of phenylacetate-coenzymeA ligases from *Burkholderia cenocepacia* (UniProt: B4E7B5) (Figure 5A), exhibits structural loss at the N-terminus with an atrophy score of 0.16 corresponding to a loss of 67 amino acid residues.

Phenylacetate-coenzymeA ligases are adenylate-forming enzymes, which are involved in the metabolism of phenylacetate [19]. The enzyme links the phosphoryl moiety of AMP to the carboxyl group of the substrate thereby activating it before transferring to the acceptor CoA [19].

The AMP-binding domain is present in proteins such as acyl-CoA synthetases [20], aryl-CoA ligases [21], firefly luciferases [22] and non-ribosomal peptide synthetases [23]. The N-terminal AMP-binding domain of the reference structure, 4-chlorobenzoyl CoA ligase from *Alcaligenes sp.* (PDB: 3CW9), shows a large, globular, distorted α/β structure comprising three distinct β-sheets with a large cleft containing the binding pocket [24] (Figure 5B).

Structural superposition of the ‘N-terminal microdomains’ of *B. cenocepacia* (PDB: 2Y27) [25] onto the reference structure (PDB: 3CW9) reveals a marked absence of the first sub-domain (residues 1-150) (Figure 5C).

It can be seen from the reference structure that the first sub-domain has minimal interactions with the substrates during adenylation and the subsequent thioester formation processes [24]. The homo-dimeric complex of *B. cenocepacia* phenylacetate-coenzymeA ligase results in the formation of an intramolecular β-sheet at the dimer interface [25]. Interestingly this interaction, involving the second sub-domains (residues 151-322), forms an arrangement that is structurally similar to the first sub-domain [25] (Figure 5D). Again a homo-dimeric arrangement is used to ensure that residues exposed by domain atrophy are buried thereby compensating for domain atrophy. The atrophied domain of *B. cenocepacia* is thought to be functional and is involved in recruiting subsequent enzymes of the phenylacetic acid degradation pathway [25]. In addition to atrophy observed in *B. cenocepacia,* we find similar domain atrophies in the phenylacetate-coenzymeA ligase orthologue from *Bacteroides thetaiotaomicron* (UniProt: Q8AAN6, PDB: 3QOV) and the phenazine antibiotic biosynthesis protein from *Enterobacter agglomerans* (UniProt: Q8GPH0, PDB: 3HGU).

### C-terminal end-bounded atrophy

#### Ral-GTPase-activating protein domain (Pfam: PF02145)

The Ral-GTPase-activating protein (RapGAP) domain of the rat Ral-GTPase-activating subunit α-1 (UniProt: O55007) has a C-terminal atrophy score of 0.46.

The 747 amino acid residue isoform 1 has an atrophied RapGAP domain at the C-terminus with no other detectable domains at the N-terminus. The RapGAP catalytic domain of the human Rap1-GAP protein (PDB: 3BRW) was chosen as reference to infer atrophy. The atrophied rat RapGAP domain and the reference human RapGAP catalytic domain show 30% sequence identity in the aligned region.

**Figure 6:**
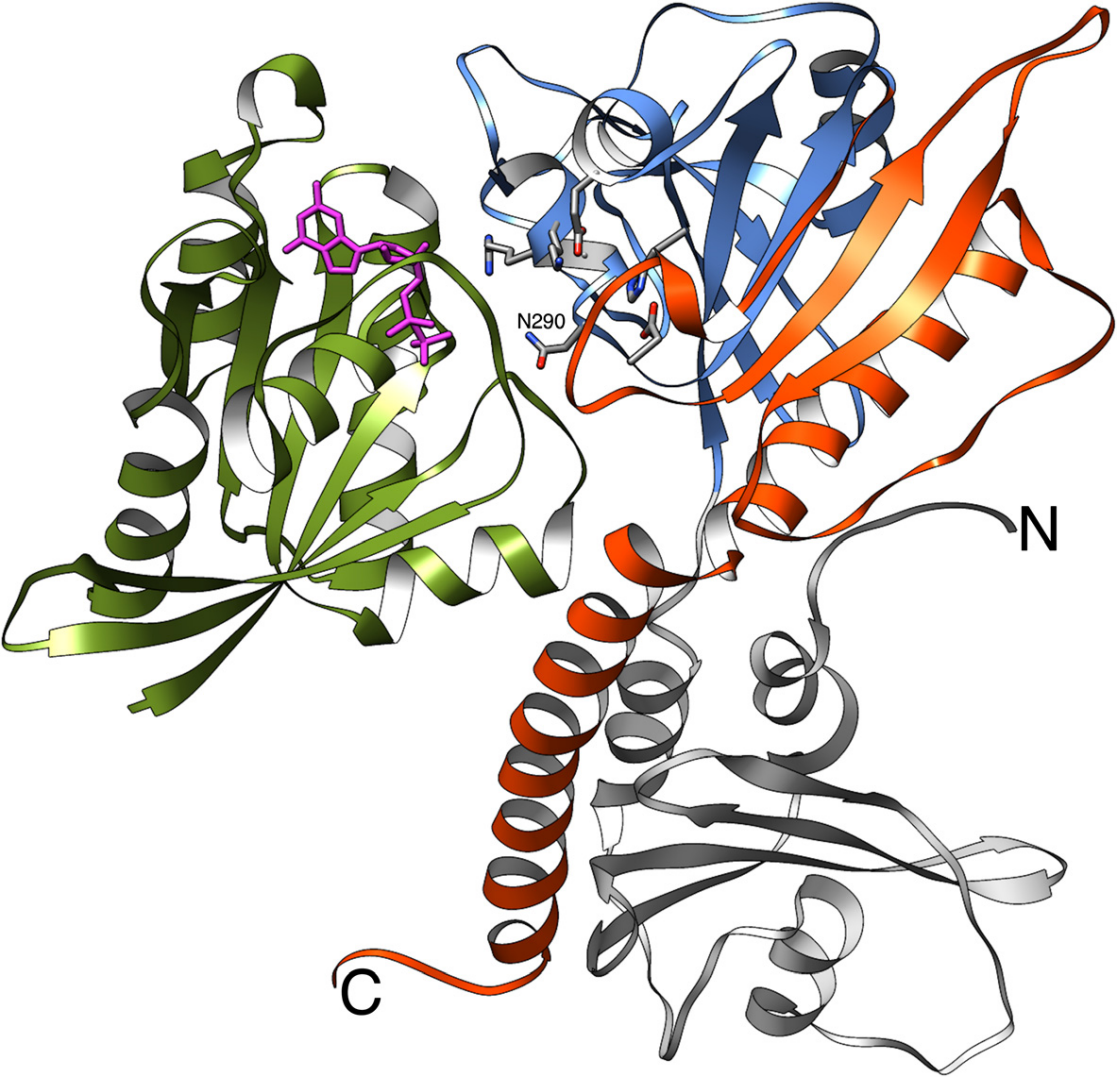
C-terminal end-bounded atrophy: Ral-GTPase-activating protein domain. The reference protein human Rap1-GAP and its interaction with Rap1B (green) bound to GDP (magenta) (PDB: 3BRW) is shown. The dimerisation domain (grey) is not involved in interaction with Rap1B. The region of atrophy of rat RapGAP domain mapped onto the reference structure is shown in orange (residues 301-414). The atrophied catalytic domain (blue) retains residues involved in catalysis or protein-interaction (grey sticks).

GTPase-activating proteins (GAPs) terminate G-protein signalling by inducing hydrolysis of bound GTP to GDP [26]. The human Rap1GAP reference protein comprises an N-terminal dimerisation and a C-terminal catalytic domain and interacts with Rap1B through the catalytic domain (Figure 6). Sequence mapping of the rat RapGAP domain onto the human Rap1-GAP protein (PDB: 3BRW) indicates structural loss in the catalytic domain (residues 301-414). The catalytic domain is an α/β structure with mixed parallel/antiparallel arrangement of β-strands and a conserved C-terminal alpha helix. The catalytic centre comprising Asn290 is close to the nucleotide-binding region and the protein interface [27,28]. The atrophy does not affect the catalytic centre or residues involved in Rap1B interaction suggesting the atrophied domain may be functional.

Rat RapGAP protein isoform 2 (906 amino acids) and isoform 3 (2,035 amino acids), however, show no signs of atrophy, suggesting exon loss mediated by alternative splicing as a probable mechanism for the observed atrophy in isoform 1.

### Upstream domain-bounded atrophy

Of the 125 cases of upstream domain-bounded atrophy identified by our pipeline none were confirmed as true atrophy events.

### Downstream domain-bounded atrophy

#### 2-hydroxyacid dehydrogenase, NAD binding domain (Pfam: PF02826)

**Figure 7:**
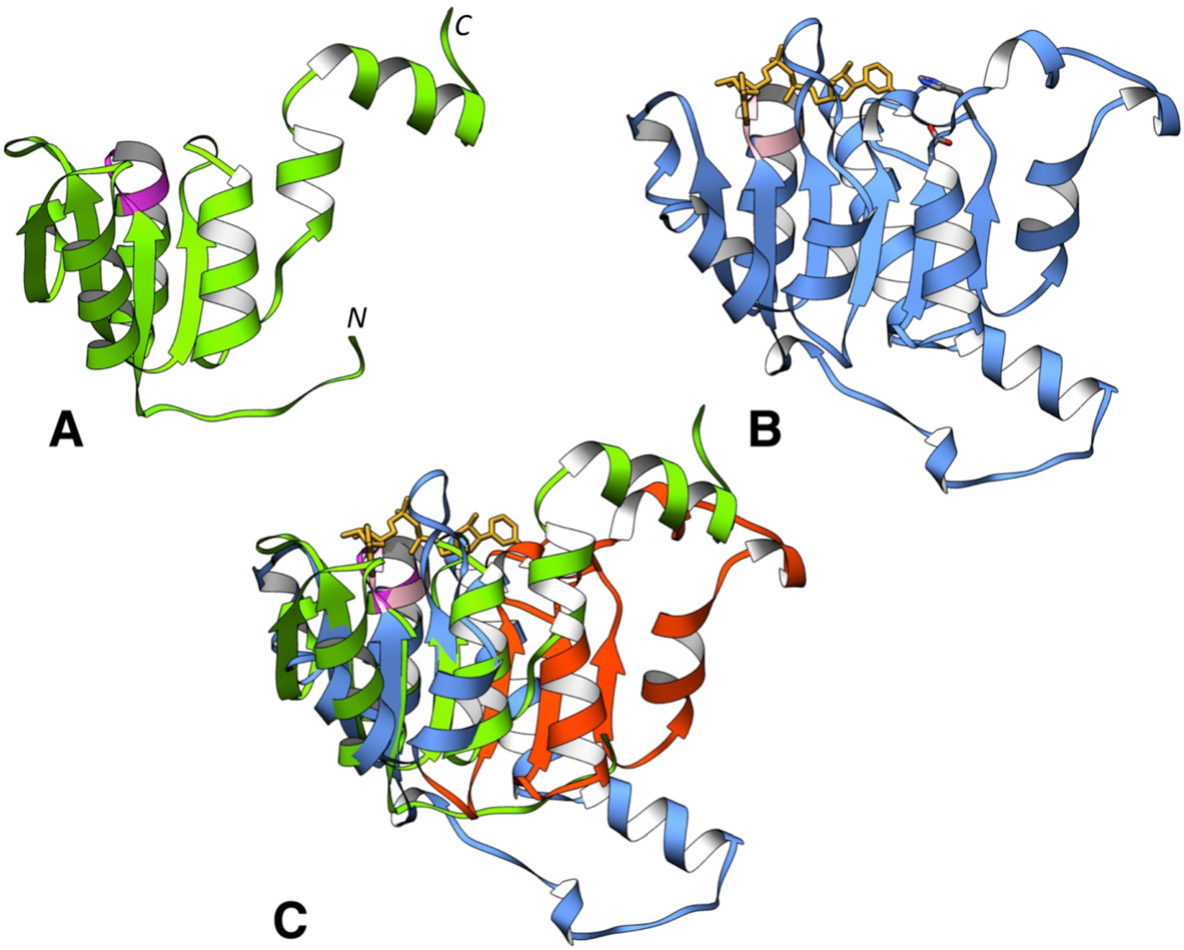
Downstream domain-bounded atrophy: 2-hydroxyacid dehydrogenase, NAD-binding domain. 2-Hacid_dh_C domains from **(A)** *Staphylococcus aureus* PurK (PDB: 3ORQ) and **(B)** *Lactobacillus jensenii* D-lactate dehydrogenase (PDB: 4PRL) **(C)** Superposition of *S. aureus* and *L. jensenii* 2-Hacid_dh_C domains shows the atrophied region (orange, residues 225-298) at the C-terminus. The reverse Rossmann-fold binding motif, GXXGXG, is conserved in both 4PRL (light pink) and 3ORQ (magenta). Note that the catalytically important Histidine (grey) that interacts with the nicotinamide moiety of NAD (gold) at the C-terminus is absent in 3ORQ.

The 2-hydroxyacid dehydrogenase NAD-binding (2-Hacid_dh_C) domain of *Staphylococcus aureus* PurK (UniProt: A6QFS4) (Figure 7A) shows an atrophy score of 0.23.

2-Hacid_dh_C domains are found in dehydrogenases and oxidoreductases among prokaryotes and eukaryotes. Bacterial PurK and PurE are involved in the two-step conversion of 5-aminoimidazole ribonucleotide to 4-carboxy-5-aminoimidazole ribonucleotide [29].

The reference 2-Hacid_dh_C domain from *Lactobacillus jensenii* D-lactate dehydrogenase (PDB: 4PRL) adopts a Rossmann fold with six parallel β-strands bound to NAD [30] (Figure 7B). The *S. aureus* (PDB: 3ORQ) 2-Hacid_dh_C domain adopts a partial Rossmann fold and have an additional alpha helix (α_2_) and β-strand (β_3_) insertion [29].

Structural superposition on 4PRL indicates a partial domain at the C-terminus of 3ORQ (Figure 7C). The atrophied domain with four parallel β-strands and four α-helices superimposes well onto the N-terminus of 4PRL, with β-strands β_2_-β_1_-β_4_ aligning on β_2_-β_1_-β_3_ of the reference domain, respectively, but has no structurally equivalent residues for β_4_-β_6_ of the reference domain (residues 225-298).

It could also be seen that despite atrophy at the C-terminus, the domain retains the ‘reverse’ Rossmann fold motif (GXXGXG) on the loop, at the N-terminus, connecting β_1_ with α_1_. This binding motif and other interaction sites are conserved and are at structurally equivalent locations (3ORQ: 16-21, 38-41) compared to the NAD bound 2-Hacid_dh_C of *L. jensenii* (4PRL: 153-158, 175-178) and might bind to the adenine moiety, however other residues interacting with the nicotinamide moiety at the C-terminus (4PRL: Asp259 and His295) are absent. His295 located near the nicotinamide ring is thought to be catalytically important [30,31] but is absent in the atrophied domain. The 2-Hacid_dh_C domain of *Bacillus anthracis* (UniProt: C3PBM5, PDB: 3Q2O) also shows similar atrophy.

#### RNase_E_G domain (Pfam: PF10150)

The RNase E domain of *Pyrococcus furiosus* RNA-binding protein AU-1 (UniProt: Q8U4Q7) shows atrophy at the C-terminus with a score of 0.19.

The *P. furiosus* RNA-binding protein is a large oligomeric complex that binds specifically to AU-rich RNA sequences and may be involved in RNA metabolic processes [32]. The RNA-binding protein AU-1 has a large conserved N-terminal RNase E domain, which shares 25% sequence similarity with *E. coli* RNase E [32], and a C-terminal domain of unknown function DUF402. There are no experimental structures available of the *P. furiosus* RNase E domain and therefore the region of atrophy was inferred through sequence mapping onto the reference structure.

**Figure 8:**
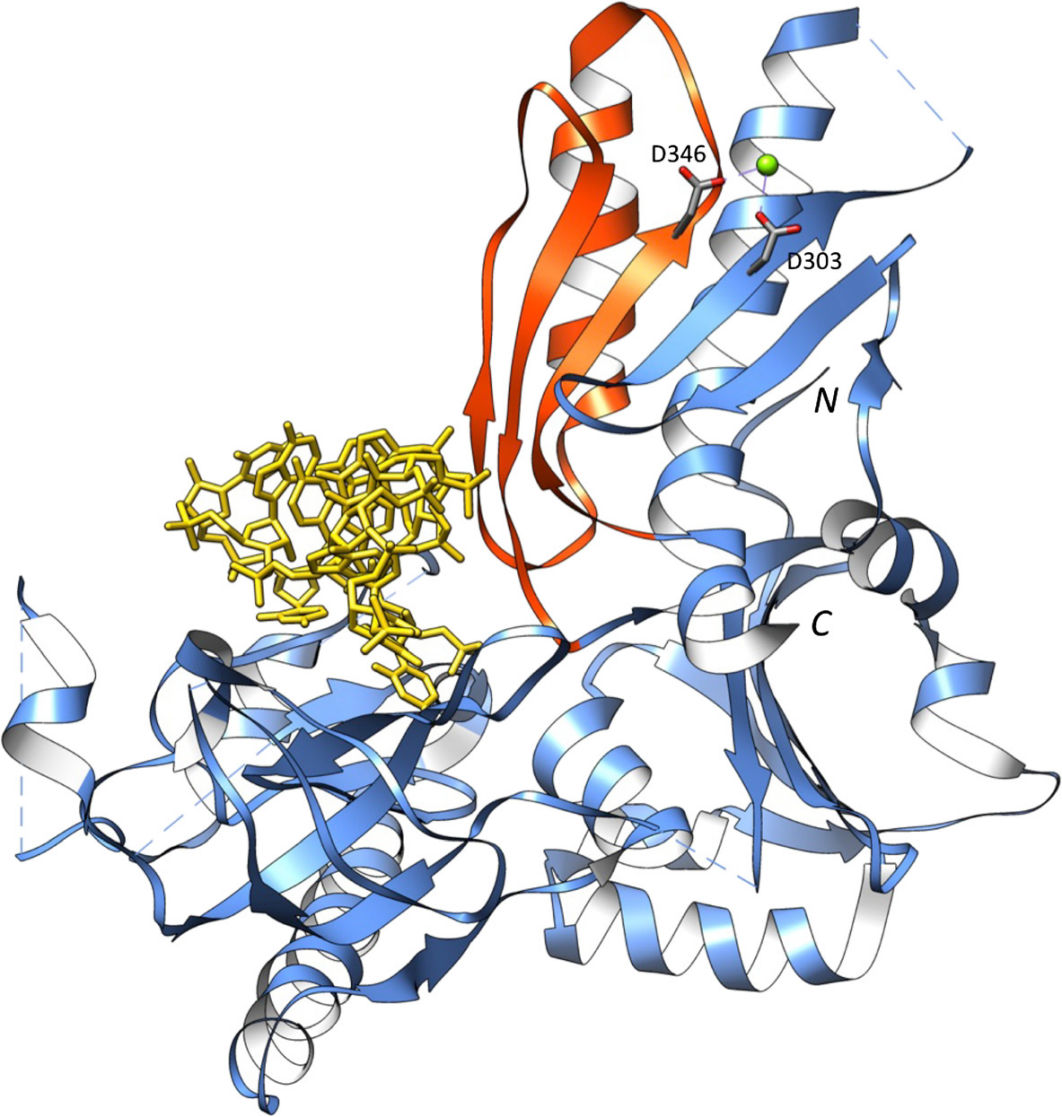
Downstream domain-bounded atrophy: RNase E large domain. The *E. coli* RNase E large domain bound to a single strand RNA (yellow) (PDB: 2C0B). The large domain comprises four different sub-domains. The atrophied region of *P. furiosus* RNase E large domain (orange, residues 339-393), inferred through sequence mapping, corresponds to nearly half of the DNase I sub-domain. The active site residues involved in cleaving RNA are shown as sticks (grey) coordinating a Magnesium ion. Missing loop regions are represented as dashed lines.

The *E. coli* RNase E large domain (PDB: 2C0B) is a large multi-domain structure comprising four domains: S1, 5’ sensing region, RNase H and DNase I [33]. Mapping of the *P. furiosus* RNase E large domain sequences on to the *E. coli* RNase E reference domain shows the region of atrophy corresponds to the C-terminus of the DNase I domain (residues 339-393) (Figure 8).

The DNase I domain has an α/β arrangement of two α-helices and an antiparallel β-sheet with six antiparallel β-strands in the order β_1–2–3–4–6–5_. The DNase I domain is the catalytic centre of the complex with active sites Asp303 and Asp346 present on β_3_ and β_4_, respectively [33]. Asp303 and Asp346 coordinate a Magnesium ion, which, through nucleophilic attack, cleaves the scissile phosphate on the RNA backbone [33]. Pairwise sequence alignment indicates *E. coli* and *P. furiosus* RNase E large domains share 14% sequence identity and atrophy at the C-terminus of the DNase I domain results in the loss of one of the active site residues, Asp346. However, a more accurate multiple sequence alignment of the domain family indicates that both catalytic residues may be present resulting in a potentially functional domain.

The *E. coli* RNase E catalytic domain is a tetrameric complex formed by a dimer-of-dimers, where the dimer interface of this complex is largely mediated by the interaction of DNase I domains [34], however, *P. furiosus* RNA-binding protein AU-1 forms a homo-oligomeric complex that is mainly trimeric [32]. The atrophy of DNase I sub-domain and the presence of a much larger C-terminal domain DUF402, compared to the *E. coli* RNase E small domain, is thought to cause the difference in the oligomerisation of the complex.

### Within-domain atrophy

#### Glycosyl hydrolase family 10 (Pfam: PF00331)

The Glycosyl hydrolase family 10 (Glyco_hydro_10) domain of *Cryptococcus albidus* Endo-1,4-beta-xylanase (UniProt: P07529) is found with a within-domain atrophy score of 0.24.

Xylanase are found in bacteria, fungi and other microbes [35,36], which degrade hemicellulose by breaking down beta-1,4-xylan into xylose. *C. albidus* endo-1,4-beta-xylanase is an inducible extracellular enzyme with xylobiose as its natural inducer [37].

**Figure 9:**
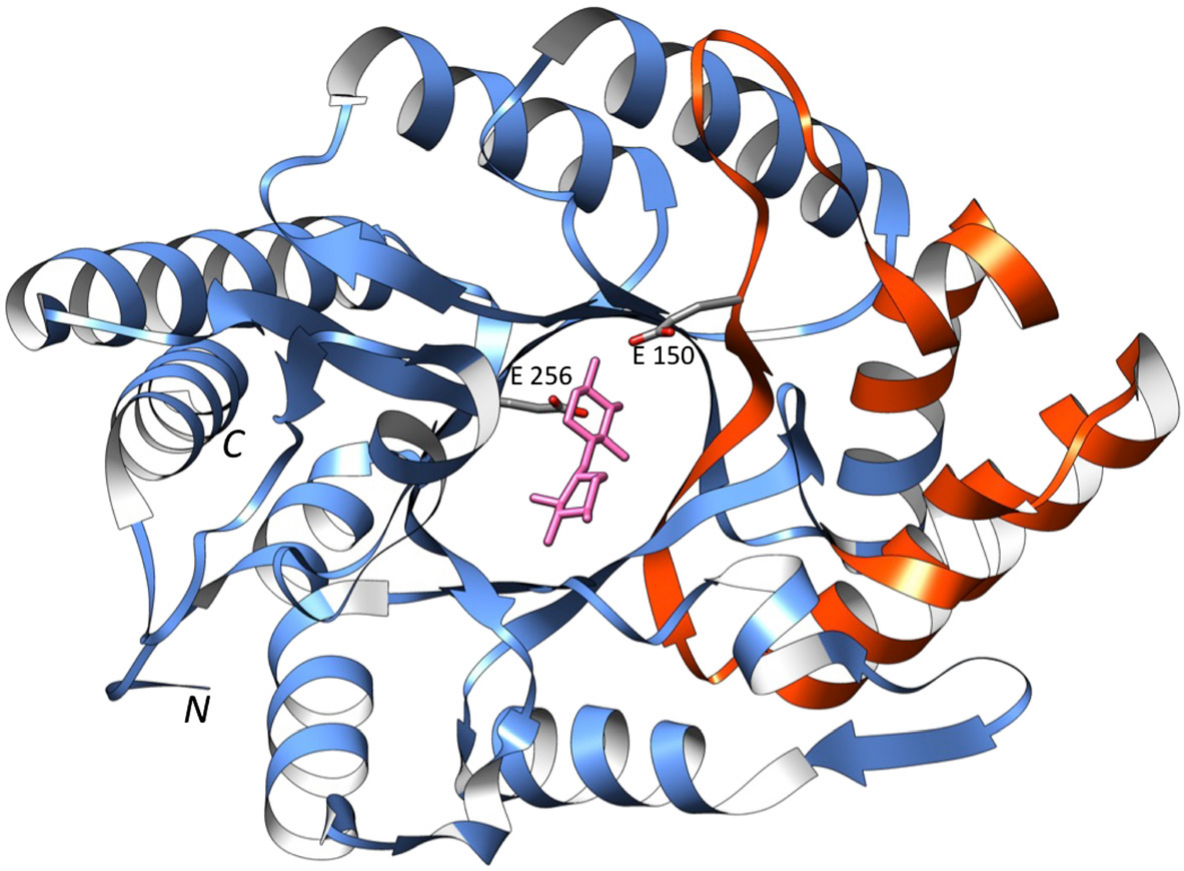
Within-domain atrophy: Glycosyl hydrolase family 10. The Glycosyl hydrolase family 10 domain of *Thermotoga petrophila* endo-1,4-beta-xylanase reference protein bound to xylobiose (pink) (PDB: 3NJ3) folds into a TIM-barrel structure. The atrophied region of *C. albidus*, inferred through sequence mapping, comprises structural elements β_4_, α_3_ and α_4_ (orange, residues 110-176) and would lose one of the active site residues Glu150 (grey).

The Glyco_hydro_10 domain of *Thermotoga petrophila* endo-1,4-beta-xylanase reference protein (PDB: 3NJ3) folds into a TIM-barrel structure [38]. Sequence mapping onto the reference domain indicates a loss of β-strand β_4_ and two core alpha helices, α_3_ and α_4_ (residues 110-176) (Figure 9). Atrophy of the *C. albidus* Glyco_hydro_10 domain would result in the loss of one of the active sites Glu150, which is present on β_4_ along with Asn149, which interacts with the ligand xylobiose.

It has been shown that *C. albidus* endo-1,4-beta-xylanase is inefficient in degrading xylobiose and hydrolyses xylotriose at very slow rates although longer substrates such as xylotetraose are hydrolysed [37,39]. It is surprising that the loss of one of the catalytic residues Glu150 could still make the enzyme functional, albeit with a reduced efficiency. Other open TIM-barrels comprising of only seven of the eight β-strands are observed in the C-terminal domains of yeast nicotinate phosphoribosyltransferase (PDB: 1VLP) and *Pyrococcus furiosus* Nicotinate-nucleotide pyrophosphorylase (PDB: 2I14). The hydrophobic cores of these partial TIM-barrels are buried by interactions with their N-terminal domains. It would be very interesting to see the structure of *C. albidus* endo-1,4-beta-xylanase to better understand how the atrophy event is compensated for.

## Conclusions

Proteins evolve by a number of different mechanisms that lead to changes in sequence, structure and function. Events such as domain gain or loss have been extensively studied in terms of evolution of prokaryotic and eukaryotic genomes [40,41], other minor events such as gain or loss of secondary structural elements, which cause structural variations in the domain, are less well studied. Domain loss has been observed as a major event during eukaryotic evolution and interestingly hints at an inverse trend with organismal complexity [40]. Unlike domain loss wherein a complete domain is absent, we have investigated events of significant structural loss occurring in a domain, by analysing variation of domain boundaries across 14,831 domain families in Pfam 27.0.

Domain atrophy events were classified into five groups based on the location of the domain within the protein and the region of atrophy in the domain. Domain atrophy events are found to be rare accounting for only a small fraction (0.06%) of the total domain instances analysed. One of the reasons for such rare occurrence is that significant loss of structural elements would usually be detrimental for domain stability. A large number of factors such as hydrophobic [42] and electrostatic interactions [43] influence the native fold and stability of the domain, and unlike domain elaborations, loss of core structural elements involved in such interactions would not be favourable.

An intriguing aspect of atrophied domains is the energetics of their folds. Deletions or insertions of individual residues can significantly influence the overall energetics of the fold [44]. Deletion of structural elements, as observed in atrophied domains, would contribute to significant changes in the energetics compared to the native fold and as such warrant major compensatory mechanisms to stabilise fold. It was shown that hydrophobic contacts at the domain-domain interface stabilise the otherwise unstable domains in a multi-domain protein [45]. Formation of multimeric complexes or domain-domain interactions may serve as one of the compensatory mechanisms for stabilising atrophied domains as observed in the cases of *P. leiognathi* bacterial luciferase domain and the AMP-binding domain of *B. cenocepacia*.

Atrophied domains should have incomplete/partial folds, but the converse may not be true. For example, the histidine biosynthesis pathway enzymes HisA and HisF consist of two copies of a (β/α)_4_ half-barrel [46]. It has been proposed that the complete (β/α)_8_ TIM-barrel may have evolved from duplication and gene fusion of an ancestral (β/α)_4_ half-barrel [46].

An important issue is to understand how domain atrophy events arise. Mutational events such as a mutation to a premature stop codon could lead to domains with atrophy at the C-terminus. While the creation of a new downstream start codon could lead to domains with atrophy at the N-terminus. Other mechanisms such as exon loss or deletion could additionally lead to atrophy within the domain. We observe a large number of domain atrophy cases at the termini of proteins: N-termini (n = 40) and C-termini (n = 26), compared to atrophy cases within a protein. We see just four cases of atrophies within a domain, and we only see five cases of atrophy events that are bounded by other domains, and hence also occur within the protein. It is unclear whether this is a reflection of the true proportions or simply due to our methodology. Alternative splicing provides one possible mechanism that enables an organism to explore atrophied domain sequence space while still retaining a functional copy [47]. Analysis of splice variants from the ENCODE dataset indicates a large number of splice sites occur within functional domains [48], suggesting a plausible mechanism for domain atrophy. It was previously shown that alternative splicing events in human result in fewer partial domains than expected [49]. These partial domains were reported to affect a significant number of functional sites [49] and may translate into proteins with altered structures and functions [47,48]. Strong selection pressure may also play a role in the evolution of atrophied domains, selecting only atrophied domains with an important structural or functional role for the organism that must be kept.

Unfolded proteins or misfolded proteins are targeted by protein degradation machinery by recognising their solvent exposed hydrophobic residues. Although no clear experimental data are available from known cases of atrophied domains on their possible mechanisms to evade recognition and degradation by the protein degradation machinery, we speculate atrophied domains escape unfolding and degradation by burying their exposed solvent-accessible areas through dimerisation or complex formations.

Disordered or transiently unfolded regions are known to fold cooperatively upon contact with their binding partners [50] and evade degradation until then. Chaperones, such as BiP, maintain unfolded or misfolded proteins in a folding-competent state [51]. We think atrophied domains with solvent exposed hydrophobic residues may be similarly protected by chaperones until folded stably upon complex formation.

We observe that domain atrophy is a very rare event compared to domain loss or elaborations. Our analyses of a subset of protein sequences, with protein existence value of 1, defined by UniProt, indicates only a minute fraction of domains exhibit atrophy. An independent analysis [13] of a broader sequence space also corroborates the findings. A large percentage of the protein domains in Pfam, annotated as partial domains, are largely found to be profile-HMM alignment or sequence annotation artefacts [13]. Our work has greatly increased the number of known atrophy cases. As identified by Triant and Pearson it is very difficult to identify true cases of domain atrophy from artefacts. Availability of high quality data with refined protein domain models and sequences, together with experimental structures of such atrophied domains, can further shed light on domain atrophy and its role in evolution of protein domains. Our work presents potential domain atrophy cases for further experimentation that will lead to an improved understanding of how proteins tolerate extreme mutations.

## Methods

### Data

To identify potential cases of domain atrophy we use matches of the UniProt sequence database (release 2012_06) against the profile-HMM models from the Pfam database release 27.0 [52]. This set of matches contains 28,738,352 Pfam-A protein domain instances across 14,831 families in 18,523,877 protein sequences.

### Atrophy score

**Figure 10:**
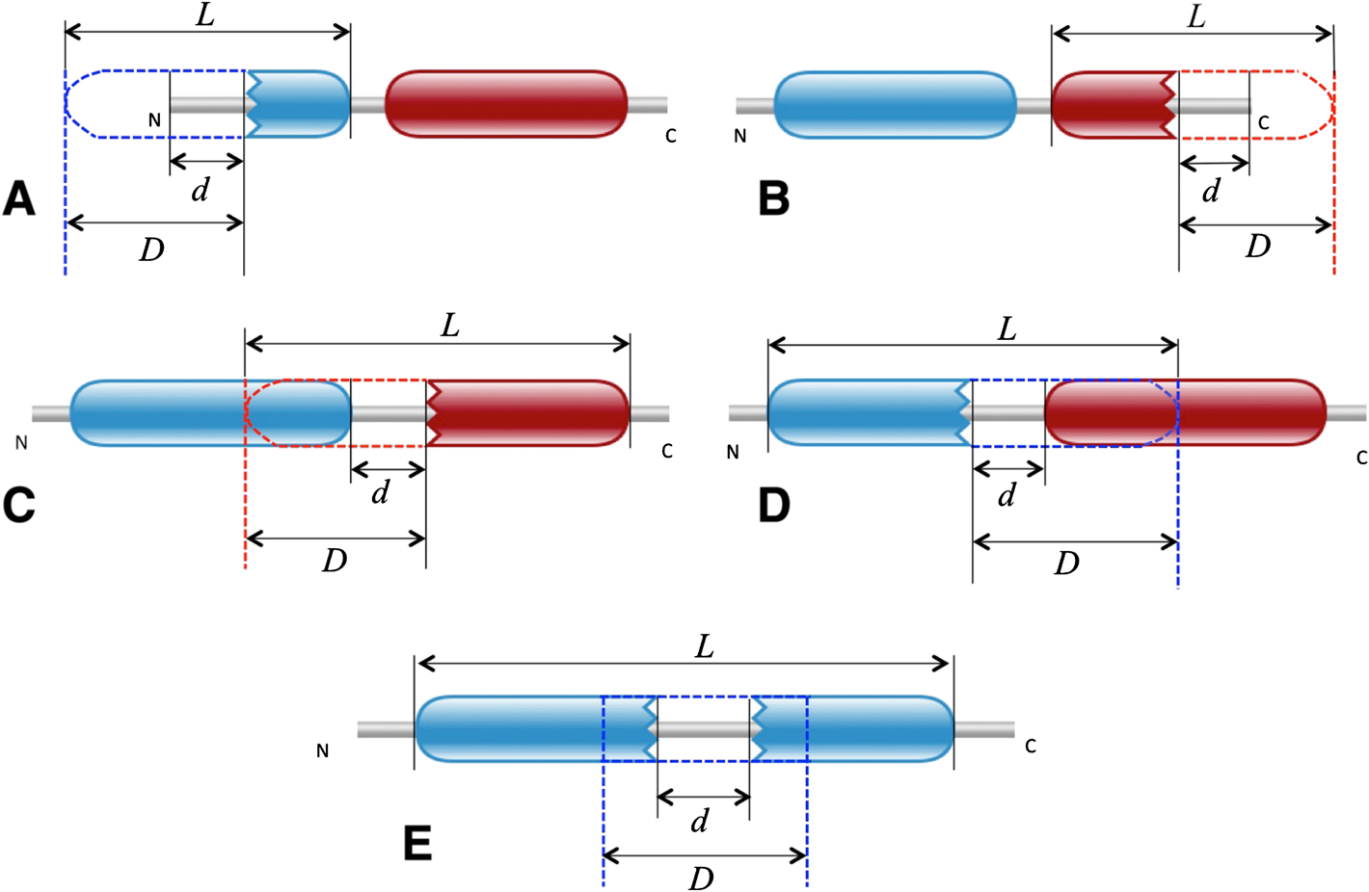
Calculation of domain atrophy score. Domain atrophy score is the ratio of missing region (*D – d*) of the domain to its model length (L). **(A)** N-terminal end-bounded atrophy. **(B)** C-terminal end-bounded atrophy. **(C)** Upstream domain-bounded atrophy. **(D)** Downstream domain-bounded atrophy. **(E)** Within-domain atrophy

To determine which domains may be cases of atrophy we calculate a measure called the Atrophy Score (AS) at both the N- (AS_N_) and C-terminal (AS_C_) boundaries of each domain instance. Figure 10 shows a schematic representation of the parameters used in calculating domain atrophy score. The equations used to calculate the atrophy score are shown below:

$$ {\mathrm{AS}}_{\mathrm{N}} = \left({\mathrm{D}}_{\mathrm{N}}\hbox{--}\ {\mathrm{d}}_{\mathrm{N}}\right)/\mathrm{L} $$

$$ {\mathrm{AS}}_{\mathrm{C}} = \left({\mathrm{D}}_{\mathrm{C}}\hbox{--}\ {\mathrm{d}}_{\mathrm{C}}\right)/\mathrm{L} $$

Where, AS_N_ is the atrophy score at the N-terminus of the domain, D_N_ is the number of unmatched HMM match states at the N-terminus of the domain, d_N_ is the inter-domain distance or domain interval that is the number of amino acid residues between the domain and its adjacent upstream domain or the sequence start site in the case of an N-terminal domain, and L is the HMM model length of the domain family. Similarly AS_C_, D_C_ and d_C_ correspond to atrophy score, the number of unmatched HMM match states and the inter-domain distance of the C-terminus of the domain, respectively.

Instances of within-domain atrophy can be identified in cases where the profile-HMM matches to a single domain have been split into two profile-HMM matches, with the first corresponding to the N-terminal part of the domain and the second corresponding to the C-terminal part of the domain. We can distinguish these cases from tandem repeats by considering the HMM match states of each domain. The start HMM-match state of the downstream domain is greater than the end HMM-match state of the upstream domain in cases of split domains. The computation of within-domain atrophy score (AS_W_) is similar to AS_N_.

$$ {\mathrm{AS}}_{\mathrm{W}} = \left({\mathrm{D}}_{\mathrm{W}}\hbox{--}\ {\mathrm{d}}_{\mathrm{W}}\right)/\mathrm{L} $$

Where D_W_ is the number of unmatched HMM match states within the domain, d_W_ is the domain interval within the domain and L is the HMM model length of the domain family.

Alignment co-ordinates of each domain are considered to calculate the inter-domain interval. An intuitive description of the Atrophy Score would be that a score of 0.33 means that one-third of the length of the domain has been lost to domain atrophy.

We classified domain atrophy events into five types, based on the domain location (architecture) in the protein and the region of atrophy in the domain: (1) N-terminal end-bounded atrophy: structural loss at the N-terminal region of the N-terminal domain; (2) C-terminal end-bounded atrophy: structural loss at the C-terminal region of the C-terminal domain; (3) Upstream domain-bounded atrophy: structural loss at the N-terminal region of an inner domain, also including the N-terminal region of the C-terminal domain; (4) Downstream domain-bounded atrophy: structural loss at the C-terminal region of an inner domain, also including the C-terminal region of the N-terminal domain; and (5) Within-domain atrophy: structural loss within the domain.

### Filtering

**Figure 11:**
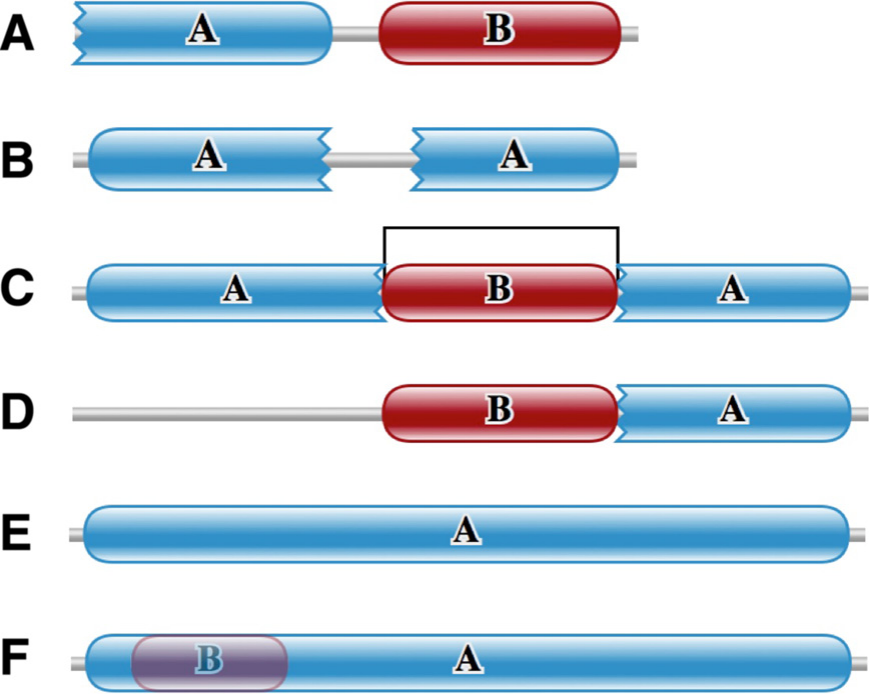
Failure modes of the pipeline. **(A)** Incorrect gene prediction or partial sequence: sequence fragment or incorrect gene prediction could lead to events that look like domain atrophy, example: Aldo/keto reductase family (UniProt: P43546, Pfam: PF00248) **(B)** Tandem repeat: a tandem repeat is distinguished from single domain instances that are split/predicted in two parts, by considering its HMM match coordinates; for a tandem domain instances the downstream domain start-HMM-match state is less than the upstream domain end-HMM-match state, example: Peroxidase (Uniprot: A0QXX7, Pfam: PF00141). **(C)** Nested domain: this architecture results in an atrophy score greater than 1 for domain hosting nested domain(s), example: Peptidase_M20 (UniProt: A0Z6B3, Pfam: PF01546) **(D)** Unmatched domain region: missing region of the domain containing a nested domain, example: Lon_C (UniProt: A4ILZ1, Pfam: PF05362) **(E)** Multi-domain family: a single-domain architecture comprising more than one domain. **(F)** Domain overlap, example: 4Fe-4S single cluster domain (UniProt: A6L094, Pfam: PF13353).

Initial results from applying the atrophy score to all UniProt proteins showed that there were numerous common failure modes (Figure 11) that would mask our ability to find genuine domain atrophy events. Therefore, we applied a set of filters to reduce the number of false positive matches with high atrophy scores.

Of a total of 23,193,494 sequences in the database, 18,523,877 sequences, which have at least one Pfam-A domain instance, were used in the analysis. Initial filtering was applied to exclude domain models from sequences with protein existence (PE) levels of 2 to 5. These are enriched in gene prediction errors and fragment proteins. This reduced the number of sequence considered from 18,523,877 to 77,305. Proteins with a protein existence level of 1 have clear experimental evidence for the existence of the protein from Edman sequencing, mass spectrometry, X-ray, NMR or other experimental evidence. Although not strictly a measure of protein sequence quality these proteins usually have highly accurate protein sequences. We also removed sequences annotated as fragments in UniProt, which further reduced the set of sequences considered from 77,305 to 75,435.

Adjacent domains that are of the same clan, similar to figure 11B, could lead to ambiguous domain boundary assignments at the interval and hence such cases were filtered out to avoid detecting false atrophy events. The resulting final-set comprising 114,303 domain instances from 75,435 sequences were included in the analysis.

Perl scripts were run to calculate atrophy scores across the set of domains. Domain instances with an atrophy score of 0.15 or more were further investigated.

### Manual inspection

Domain instances that were obtained after applying the above filters were then selected for manual inspection. Only those domains that had an atrophy score of 0.15 or above were checked manually for identification of false positives. We checked each potential domain atrophy case for evidence of any of the following failure modes (see Figure 11): (1) Gene prediction errors: We checked whether the missing part of a domain was to be found in an adjacent gene or due to an incomplete gene prediction; (2) Nested domains: We checked whether a high atrophy score was due to a domain nesting within another. These were considered as false positives; (3) Multi-domain families: Due to incorrect Pfam domain definitions some Pfam domains actually correspond to multiple structural domains that can be found independently. We checked the structure of each Pfam family to confirm whether this was the cause of a high atrophy score; (4) Small domains: Domains of length less than 30 amino acid residues were not considered since atrophy score of 0.15 and above of small domains correspond to loss of a single secondary structural element or a part thereof, which is not considered true atrophy; (5) Circular permutations: While circular permuted domains are complete domains, the rearrangement of domain HMM start-site and HMM end-site with respect to their domain HMM-model would result in misidentification of such cases as domain atrophies; (6) Short repeats: Domains composed of tandem structural motifs, such as β-propeller, β- or α-helix, are made of short repeating sequence motifs were considered as false positives. Addition or removal of repeats is often tolerated in terms of protein mutation; and (7) Disordered domains: Inferring domain atrophy among intrinsically disordered protein domains is not straightforward mainly owing to their lack of native ordered tertiary structure and such cases were considered false positives.

Apart from the above failure modes domain atrophy cases whose structures were theoretically modelled or had no other reference structures in the family to compare with were also treated as failure modes. Other cases of complete structural domains but identified as domain atrophy were treated as false positives. Table 2 lists the occurrences of different types of failure modes.

**Table 2:** **Cases of failure modes or false positives**

**Types of failure modes or false positives**	**Number of instances**
Total	1,287
Gene prediction error	9
Containing nested domain	268
Multi-domain family	316
Small domain	173
Circular permutation	54
Short repeat	112
Disordered domain	85
Theoretical model	9
No other reference structure available	27
Complete structural domain	234

Domain instances with atrophy scores between 0.15 and 1 that were manually checked and classified into one of the failure modes or false positives.

From a total of 1,636 domain instances, with atrophy scores between 0.15 and 1, which were manually checked, 1,287 domain instances were classified as failure modes or false positives. The positive predictive value (PPV) of our method is 0.055.

### Visualisation of structures

Structures were visualised with Chimera [53]. Where experimental structures of atrophied domains were not available, the shortest full-length domain structure within the domain family was chosen as the reference. The extent of domain atrophy was then inferred by a pairwise sequence alignment guided mapping of unaligned sequence regions onto the reference structure. Instances of putative domain atrophy where no full-length reference structure was available for the family were not considered further.

### Phylogenetic analysis

Evolutionary information was inferred from phylogenetic trees constructed from multiple sequence alignment of domain family seed sequences and homologous sequences from a JackHMMER search [54]. A non-redundant set of sequences of 90% identity or less was aligned with Mafft [55] with default settings. Alignments were visualised with Belvu [56] and phylogenetic trees constructed using the neighbour-joining method present in Belvu using default parameters.

## Additional file

Additional file 1:
**Supplementary data.** Domain atrophy cases: List of true and putative domain atrophy cases.
